# Layered Perovskites BaM_2_In_2_O_7_ (M = La, Nd): From the Structure to the Ionic (O^2−^, H^+^) Conductivity

**DOI:** 10.3390/ma15103488

**Published:** 2022-05-12

**Authors:** Nataliia Tarasova, Anzhelika Galisheva, Irina Animitsa, Ksenia Belova, Anastasia Egorova, Ekaterina Abakumova, Dmitry Medvedev

**Affiliations:** Institute of High Temperature Electrochemistry of the Ural Branch of the Russian Academy of Sciences, 620066 Ekatherinburg, Russia; a.o.galisheva@urfu.ru (A.G.); irina.animitsa@urfu.ru (I.A.); ksenia.belova@urfu.ru (K.B.); oav-hn@yandex.ru (A.E.); e.v.abakumova@urfu.ru (E.A.); dmitrymedv@mail.ru (D.M.)

**Keywords:** BaLa_2_In_2_O_7_, BaNd_2_In_2_O_7_, layered perovskite, Ruddlesden–Popper structure, water uptake, oxygen-ion conductivity, protonic conductivity

## Abstract

The design of new oxide compounds that can be used as oxygen- or proton-conducting electrolytes for solid oxide fuel cells is actively in progress. Despite the intensive research activities regarding electrolytes with perovskite/fluorite structures, the search for other structural alternatives is of paramount importance. In this study we focus on a novel material with significantly improved properties for the electrochemical purposes. The two-layered BaNd_2_In_2_O_7_ perovskite with a Ruddlesden–Popper structure was investigated as a protonic conductor for the first time. In detail, its local structure, water uptake, and the ionic (O^2−^, H^+^) conductivity were comprehensively studied. The nature of rare-earth elements (M = La, Nd) in the structure of BaM_2_In_2_O_7_ on the structural and transport properties was revealed. The presented analysis showed that the composition of BaNd_2_In_2_O_7_ is nearly pure proton conductor below 350 °C. This work opens up a new way in the design of protonic conductors with double-layered perovskite structure.

## 1. Introduction

**Figure 1:**
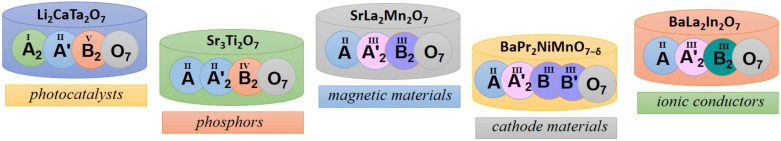
The overview of materials with a double-layered Ruddlesden–Popper structure.

The materials with layered perovskite-related structures have many various applications due to their different physical-chemical properties. These properties are dependent on the nature of ions in the crystal lattice. The structure of the compositions with the general formula ${\mathrm{AA}}_{2}^{\prime }B_{2}O_{7}$ or AO(A′BO_3_)_2_ can be described as the block-layered Ruddlesden–Popper (RP) structure where blocks consisting of two perovskite octahedra A′BO_3_ alternate with the salt AO layers [1]. The sum of charges of the cations in the A/A′- and B-sublattices can be obtain by the different variations such as +4 and +10, +6 and +8, and +8 and +6 (Figure 1). The total charge +4 in the A/A′-sublattice is typical for photocatalysts A_2_A′Ta_2_O_7_ where A is the hydrogen or alkali metal and A′ is the alkali-earth metal [2,3,4]. The phosphors such as Sr_3_Sn_2_O_7_:Eu^3+^ [5] and Sr_3_Ti_2_O_7_:Eu^3+^ [6] have total charge +6 and +8 in the A- and B-sublattices, respectively. For the magnetic A^II^Ln_2_Mn_2_O_7_ [7,8,9,10,11,12] and cathode A^II^Ln_2_MM′O_7_ [13,14,15,16] materials the sum of cationic charges can be written as +8 and +6, where A^II^ is the alkali-earth metal, Ln is the lanthanide, M and M′ are metals with variable oxidation state such as Mn, Fe, Co, or Ni. In the case of the presence in the B-sublattice the metal with constant oxidation state such as indium, the chemical properties become completely different despite the same +8/+6 sum of cationic charges. As it was shown recently [17], the composition BaLa_2_In_2_O_7_ demonstrates nearly pure protonic transport under wet air and low temperatures and this phase can be potentially considered as the electrolytic material for the solid oxide fuel cell; therefore, the development of new materials with improvement properties is very relevant today [18,19,20,21,22,23,24,25,26].

The structure of the compounds BaM_2_In_2_O_7_ (M = La, Nd) was described earlier by Titov et al. [27] and Raveau et al. [28]. It was shown that these phases belong to the RP structure AO(A′BO_3_)_n_ where *n* = 2. The compositions with monolayered (*n* = 1) RP structure in the system Ba-Ln-In-O are exist also. The complex oxides BaNdInO_4_ [29,30,31,32,33,34] and BaLaInO_4_ [35,36,37,38,39,40,41] and doped compositions based on it are mixed ionic-electronic or protonic conductors depending on the water partial pressure in the atmosphere and temperature. At the same time, in the row BaLaInO_4_—BaNdInO_4_ the ionic conductivity increased. Based on this, it can be predicted the same dependency in the row BaLa_2_In_2_O_7_–BaNd_2_In_2_O_7_. From this point of view, the phase BaNd_2_In_2_O_7_ is of interest for studying their physicochemical properties.

In this paper, the local structure, water uptake, and the ionic (O^2−^, H^+^) transport in the complex oxide BaNd_2_In_2_O_7_ were investigated for the first time. It was shown that this material can be considered as the promising matrix compositions for development of novel high-conductive protonic electrolytes with PR structure.

## 2. Materials and Methods

The complex oxides BaNd_2_In_2_O_7_ and BaLa_2_In_2_O_7_ were obtained by the method of solid-state synthesis. The powder initial materials BaCO_3_, La_2_O_3_, Nd_2_O_3_, and In_2_O_3_ were previously dried, weighed, and mixed in stoichiometric quantities. The milling of powders was made in agate mortar. The compositions were calcined at 800, 900, 1000, 1100, 1200, and 1300 °C for 24 h at air, intermediate grindings were made for every following heating step.

The X-ray diffraction studies were made by the Bruker Advance D8 Cu K*_α_* diffractometer (Billerica, MA, USA) with a step of 0.01° and at a scanning rate of 0.5°/min. The local structure of the samples was investigated by the WiTec Alpha 300 AR Raman microscopy system (objective lens, blue laser, Ulm, Germany). The morphology and chemical composition of the samples were studied using a scanning electron microscope Phenom ProX Desktop (SEM) (Waltham, MA, USA) integrated with energy-dispersive X-ray diffraction (EDS) detector.

The Perkin Elmer Pyris 1 TGA thermogravimetric analyzer (London, UK) was used for the investigation of thermal behavior of the hydrated phase. The heating of initially hydrated samples was made at the temperature range of 40–1100 °C with speed of 10 °C/min under a flow of dry Ar. The hydrated samples were obtained at slow cooling from 1100 to 150 °C (1 °C/min) under a flow of wet Ar (99.999% purity, *p*H_2_O = 2 × 10^−2^ atm). Ar atmosphere was used to avoid any carbonization of the samples.

The electrical conductivity was measured on the pressed cylindrical pellets using impedance spectroscopy method. The impedance spectrometer Z-1000P (Elins, RF, Uppsala, Sweden) with the frequency range of 1–10^6^ Hz was used. The dependencies of conductivities vs. temperature were obtained in the temperature range of 200–1000 °C (step 10–20 °C, 1°/min cooling rate). All electrochemical investigations were performed under dry and wet air or Ar. The dry gas was produced by circulating the gas through P_2_O_5_ (*p*H_2_O = 3.5 × 10^−5^ atm). The wet gas was obtained by bubbling the gas at room temperature first through distilled water and then through saturated solution of KBr (*p*H_2_O = 2 × 10^−2^ atm). The humidity of the gas was controlled by a Honeywell HIH-3610 H_2_O sensor. The dependencies of conductivities vs. partial oxygen pressures *p*O_2_ were obtained by using the electrochemical method for producing different *p*O_2_ with oxygen pump (and sensor) from Y-stabilized ZrO_2_ ceramic. The values of the resistance were recorded after 3–5 h of equilibrium.

## 3. Results

### 3.1. Material Characterization

**Figure 2:**
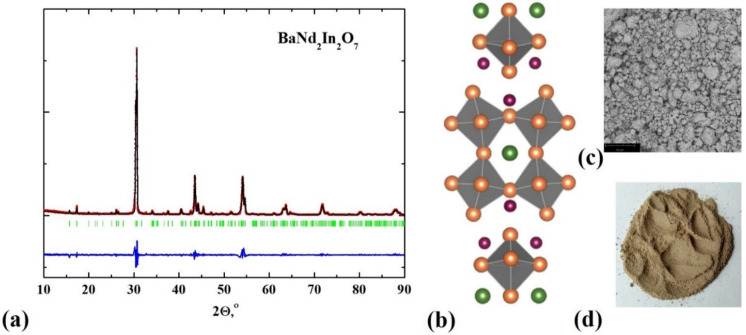
Materials characterization of the BaNd_2_In_2_O_7_ compound: XRD patterns (**a**), image of double-layered RP crystal structure (**b**), SEM-image (**c**), and image of powder sample (**d**).

The powder samples BaM_2_In_2_O_7_ (M = La, Nd) were investigated using X-ray diffraction analysis. The both compositions were single phase, and they are indexed in the tetragonal symmetry (space group *P*4_2_/*mnm*) (Figure 2a). The lattice and structural parameters were in good agreement with previously reported by Titov et al. and Raveau et al. data [27,28] (Table 1).

The morphology of the samples was investigated using scanning electron microscopy (SEM) method. The size of the grains was ~5–10 μm; the agglomerates with the size up to 30–50 μm were also found (Figure 2c). Figure 2d represents the image of the powder sample BaNd_2_In_2_O_7_. The chemical composition was checked via the energy-dispersive (EDS) analysis. The good agreement between theoretical and experimental values of chemical composition was proved by the energy-dispersive (EDS) analysis performed on the polished cleavages of the ceramic samples (Table 2).

### 3.2. Oxygen-Ionic Conductivity

**Figure 3:**
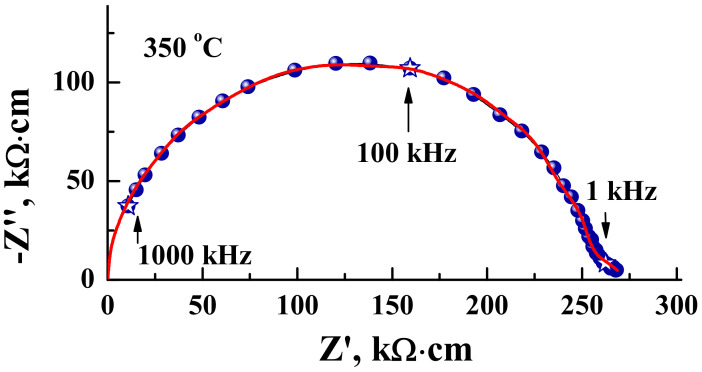
The Nyquist-plot for the BaNd_2_In_2_O_7_ ceramic material obtained at 350 °C under dry air.

The conductivity measurements were made using the impedance spectroscopy method. As an example, the typical Nyqiust plot is presented in Figure 3. The experimental data are showed by the blue symbols, and the fitting of the spectra (ZView software) is represented by the red line. According to the spectra, two different electrochemical processes can be defined. The semicircle started from the zero coordinates corresponds to the bulk resistance with the capacity ~10^−12^ F. The small semicircle in the low-frequency region is characterized by the capacity ~10^−10^ F and it corresponds to the resistance of the grain boundaries. It should be noted what the Nyqiust plots for the monolayer composition BaNdInO_4_ described by Yang et al. [33] was characterized by the same shape. For the calculation of electrical conductivity, the bulk resistance values were used.

**Figure 4:**
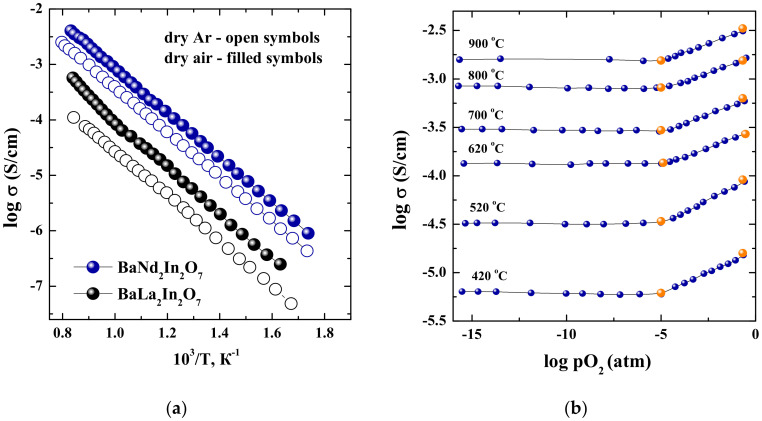
Temperature dependencies of conductivity for BaNd_2_In_2_O_7_ (blue symbols) and BaLa_2_In_2_O_7_ (black symbols) [17] obtained for dry Ar (open symbols) and dry air (filled symbols) (**a**); jxygen partial pressure dependencies of the total conductivity values for the BaNd_2_In_2_O_7_ sample at dry conditions (blue symbols) and conductivity values from *σ*—10^3^/*T* dependencies in dry air and Ar (orange symbols) (**b**).

The temperature dependencies of conductivity obtained under dry condition (*p*H_2_O = 3.5 × 10^−5^ atm) are presented in Figure 4a. As can be seen, the conductivity values for the composition BaNd_2_In_2_O_7_ are higher than for the BaLa_2_In_2_O_7_ [17]. The increase in the conductivity is about one order of magnitude under dry air condition. In the dry Ar, this difference is up to 1.2 order of magnitude. The dependencies of conductivity vs. oxygen partial pressure under dry condition are presented in Figure 4b. The right part of the curves (in the *p*O_2_ range of 10^−5^–0.21 atm) had a positive slope which corresponded to the electronic conductivity (p-type). The conductivity values are independent on the oxygen partial pressure below 10^−5^ atm, and this region belongs to the electrolytic area. It should be noted, that conductivity values obtained from “σ–1/T” dependencies (orange symbols, Figure 4b) well correlated with the values obtained from “σ–*p*O_2_” dependencies (blue symbols, Figure 4b). Based on this, the conductivity values obtained under dry Ar can be considered as the oxygen-ionic conductivity values. Consequently, the oxygen-ionic transport number $t_{O^{2-}}$ can be calculated as:

(1) $$t_{O^{2-}}=\frac{\sigma_{O^{2-}}}{\sigma_{\mathrm{tot}}}=\frac{\sigma_{\mathrm{Ar}}}{\sigma_{\mathrm{air}}}$$

The oxygen-ionic transport numbers for the compounds BaLa_2_In_2_O_7_ and BaNd_2_In_2_O_7_ were around 20% and 50% correspondingly, i.e., the share of oxygen transport was bigger for the Nd-containing sample. For the explanation of this fact, the nature of oxygen transport should be considered.

The layered RP-structure BaM_2_In_2_O_7_ with *n* = 2 consists of alternating salt layers and perovskite blocks where two octahedra [InO_6_] layers are connected to each other by vertices (Figure 2b). The barium atoms are located in the perovskite blocks in the space between [InO_6_] octahedra and they have 12 coordination numbers. The atoms of rare earth elements (La and Nd) are placed in the salt layers and they characterized by a coordination number of 9. The increase in the coordination number of La or Nd atoms up to 12 is theoretically possible, and this process can be described in terms of anti-Frenkel disordering:

(2) $$O_{O}^{x}\Leftrightarrow V_{o}^{••}+O_{i}^{\prime \prime }$$

where $O_{i}^{\prime \prime }$ is the oxygen atom in the interstitial position; $V_{O}^{••}$ is the oxygen vacancy. In this case, the coordination number of La/Nd atoms increase, and the coordination number of In atoms decrease. Returning to the experimental data, we can say, that in the row BaLa_2_In_2_O_7_–BaNd_2_In_2_O_7_ not only total conductivity increases, but the share of oxygen-ionic transport increases also. Consequently, the different nature of rare earth elements (La or Nd) in the crystal lattice of layered perovskite BaM_2_In_2_O_7_ leads to the changes in the degree of disordering and the change in the local structure.

**Figure 5:**
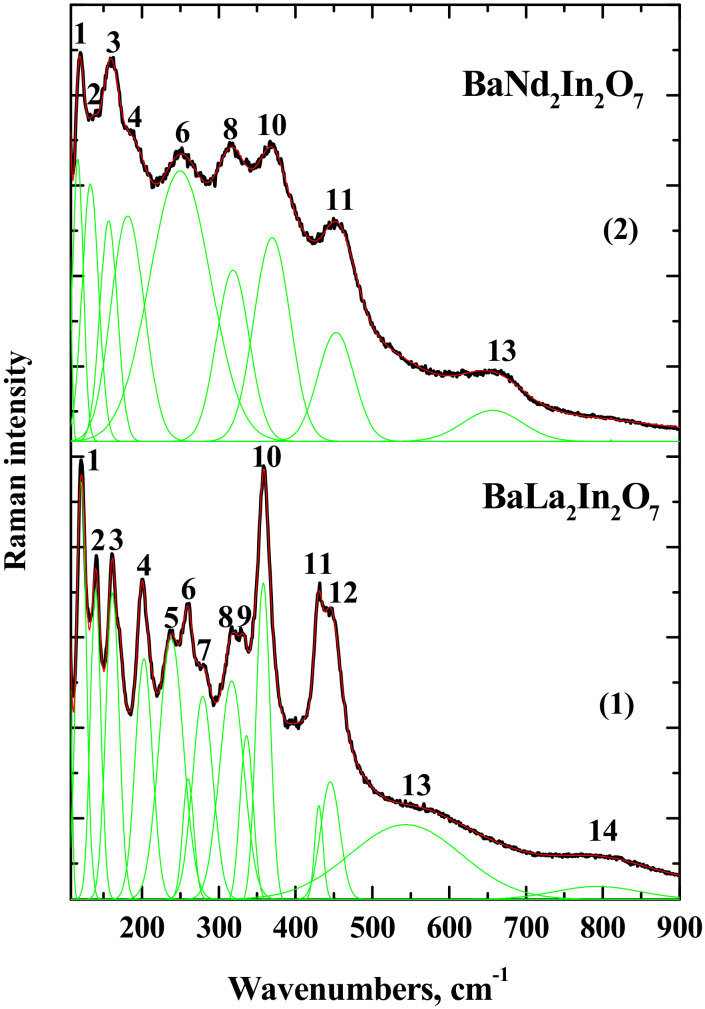
Raman spectra of the BaLa_2_In_2_O_7_ (**1**) and BaNd_2_In_2_O_7_ (**2**) compounds.

The local structure of compounds BaLa_2_In_2_O_7_ and BaNd_2_In_2_O_7_ was investigated using Raman spectroscopy. The results of the deconvolution of obtained Raman spectra are presented in Figure 5. The region of low wavenumbers (120–200 cm^−1^) is represented by the bending and stretching vibrations of polyhedral, containing alkali earth and rare earth metals [40,41,42,43], i.e., [BaO_12_], [LaO_9_], and [NdO_9_]. In this region, the modes ν_1_, ν_2_, ν_3_, and ν_4_ are observed (Table 3). They can be ascribed to the M–O stretching and O–M–O bending vibrations where M is the Ba, La, or Nd. For the composition BaNd_2_In_2_O_7_ the red shift (i.e., shift towards lower wavenumbers) is observed. In general, the red shift indicates the increase in the part of bond lengths M–O. However, due to the presence of several cations in the A- and A′-sublattices of ${\mathrm{AA}}_{2}^{\prime }B_{2}O_{7}$, the correct interpretation of this shift is difficult.

The region of mid and high wavenumbers (higher than 200 cm^−1^) contains the tilting/bending and stretching vibrations of In-contained polyhedra. The band ν_5_, ν_6_, ν_7_, ν_8_, and ν_9_ can be described as the tilting/bending vibration of polyhedra [InO_6_]. These five bands are well visible in the spectrum of BaLa_2_In_2_O_7_, while the spectrum of BaNd_2_In_2_O_7_ contains two (ν_6_ and ν_8_) bands only. It can be said, that ν_6_ and ν_8_ bands in the spectrum of BaNd_2_In_2_O_7_ degenerate to the ν_5_, ν_6_, ν_7_, ν_8_, and ν_9_ bands in the spectrum of BaLa_2_In_2_O_7_ correspondingly. Based on this, it can be assumed that tilting of In-containing octahedra decreases in the row BaLa_2_In_2_O_7_–BaNd_2_In_2_O_7_. According to the structural data reported by Titov et al. [27], the degree of deformation of [InO_6_] polyhedra decreases and the In–O–In angle is approaching to 180° from BaLa_2_In_2_O_7_ to BaNd_2_In_2_O_7_. The band ν_14_ registered in the spectrum of BaLa_2_In_2_O_7_ can be ascribed to the repulsion between the Ba^2+^/La^3+^(Nd^3+^) ions and apical oxygen ions in compressed In-contained polyhedra [44]. The decrease in the tilting of In-contained polyhedra in the row BaLa_2_In_2_O_7_–BaNd_2_In_2_O_7_ can lead to the disappearance of this band in the spectrum of BaNd_2_In_2_O_7_.

The stretching vibrations of In-contained polyhedra locate in the higher wavenumbers. Based on the analysis of Raman spectra of monolayer RP-composition BaLaInO_4_ [40,41], the stretching vibrations of In–O bonds should be appeared around 400 cm^−1^. At the same time, the comparable analysis of Raman spectra of RP homologous series Sr_n+1_Ti_n_O_3n+1_ [45] and Sr_n+1_Ru_n_O_3n+1_ [46] showed that the signal corresponded to the M–O stretching vibrations for monolayer compositions transforms into two signals with lower and higher wavenumbers in the spectra of double-layered RP-compositions. Thus, the bands ν_10_, ν_11_, and ν_12_ can be attributed to stretching vibrations of In–O bonds. As can be seen, the blue shift (i.e., shift towards higher wavenumbers) of ν_10_ and ν_11_ bands is observed for BaNd_2_In_2_O_7_ composition compared with BaLa_2_In_2_O_7_ composition. This shift indicates the decrease in some In–O bond lengths.

The local distortion of the crystal lattice due to anti-Frenkel disordering (Equation (2)) can lead to the formation of oxygen vacancies in the In-contained polyhedra, and the polyhedra with lower coordination number [InO_6−x_] appear in the structure. At the same time, the formation of oxygen interstitial leads to the increase in the coordination number of some cations. Most reasonable candidate for this is the rare earth element because both La and Nd have a coordination number of 9, and they can increase it up to 12 theoretically. Thus, the formation of polyhedra [La(Nd)O_9+x_] is possible. It is obvious that the decrease in the coordination number of metal leads to the decrease in some part of metal-oxygen bond length, and the signal with higher wavenumber should appear in the spectrum, what was observed for the composition BaLaInO_4_ [40,41]. We can assume that the signal ν_13_ in the spectra of BaNd_2_In_2_O_7_ and BaLa_2_In_2_O_7_ can be attributed to the motion of In-contained polyhedra with a lower coordination number.

The blue shift of ν_13_ band for the BaNd_2_In_2_O_7_ indicates that the decrease in the part of In–O bond lengths is more pronounced. Thus, we can assume that for the Nd-contained compound the formation of oxygen vacancies due to local distortion is more favorable in comparison with BaLa_2_In_2_O_7_ composition. This result is in good agreement with the crystallographic point of view, because the lower coordination number is more favorable for the ion with smaller ionic radius ($r_{{\mathrm{Nd}}^{3+}}$ = 1.163 Å, $r_{{\mathrm{La}}^{3+}}$ = 1.216 Å [47]). In this way, the concentration of oxygen defects (oxygen vacancies and oxygen interstitial) should be higher in the composition BaNd_2_In_2_O_7_ compared with the composition BaLa_2_In_2_O_7_, and the share of oxygen-ionic conductivity should be higher also. Returning to obtained experimental data, we can say, that the results of electrical measurements are well correlated with the Raman spectroscopy results. The electrical conductivity and the share of oxygen-ionic transport increased in the row BaLa_2_In_2_O_7_–BaNd_2_In_2_O_7_.

### 3.3. Protonic Conductivity

The possibility of water uptake for the compounds with PR-structure is provided by the opportunity of the cations increase their coordination numbers. For the composition BaM_2_In_2_O_7_ the hydration may be due to increase in the coordination number of atoms M and the formation of polyhedra [MO_8_(OH)_2_]. This process can be described by the quasi-chemical equation as:

(3) $$H_{2}O+O_{o}^{x}\Leftrightarrow {(\mathrm{OH})}_{o}^{•}+{\left(\mathrm{OH}\right)}_{i}^{\prime }$$

where ${(\mathrm{OH})}_{o}^{•}$ is the hydroxyl group in the regular oxygen position; ${\left(\mathrm{OH}\right)}_{i}^{\prime }$ is the hydroxyl group located in the interlayer space. However, in the case of the presence oxygen vacancies in the structure, the water uptake can be described also as:

(4) $$V_{o}^{••}+H_{2}O+O_{o}^{x}\Leftrightarrow {\left(\mathrm{OH}\right)}_{o}^{•}+{\left(\mathrm{OH}\right)}_{V_{o}}^{•}$$

It is obvious that both of these processes are happened at the same time for the compositions with local distortion of the crystal lattice. Based on this, we can assume that in the structure of hydrated RP-compositions at least three different crystallographic positions of oxygen-hydrogen groups exist. Protons can be localized the structural oxygen atoms ${(\mathrm{OH})}_{o\prime }^{•}$ and part of the oxygen-hydrogen groups may exist in the oxygen vacancies ${\left(\mathrm{OH}\right)}_{V_{o}}^{•}$ in the perovskite blocks and in the salt block in the interstitial oxygen positions ${\left(\mathrm{OH}\right)}_{i}^{\prime }$.

**Figure 6:**
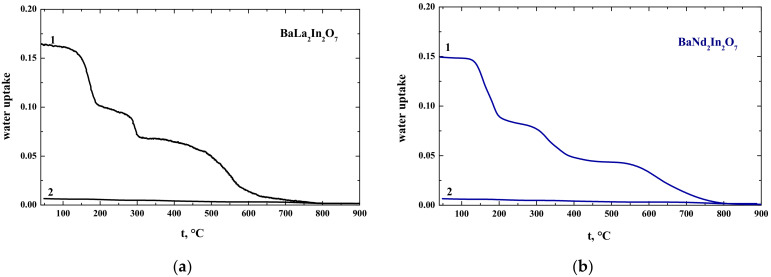
Thermogravimetry data for the BaLa_2_In_2_O_7_ (**a**) and BaNd_2_In_2_O_7_ (**b**) compounds obtained under wet (1) and dry (2) Ar.

Figure 6 represents the thermogravimetric (TG) data of hydrated compositions BaLa_2_In_2_O_7_ and BaNd_2_In_2_O_7_. As we can see, three steps on the TG-curves for both compositions are observed. These results can be indirect evidence of the presence of oxygen vacancies in the structure of compositions BaM_2_In_2_O_7_. The amount of water uptake is almost the same for both compositions and it is around 0.15–0.17 mol H_2_O per formula BaM_2_In_2_O_7_ unit. Consequently, the difference between protonic conductivity values for the compositions BaLa_2_In_2_O_7_ and BaNd_2_In_2_O_7_ must be provided by the difference between protonic mobility only:

(5) $$\sigma_{i}=z_{i}\cdot e\cdot \mu_{i}\cdot c_{i}$$

**Figure 7:**
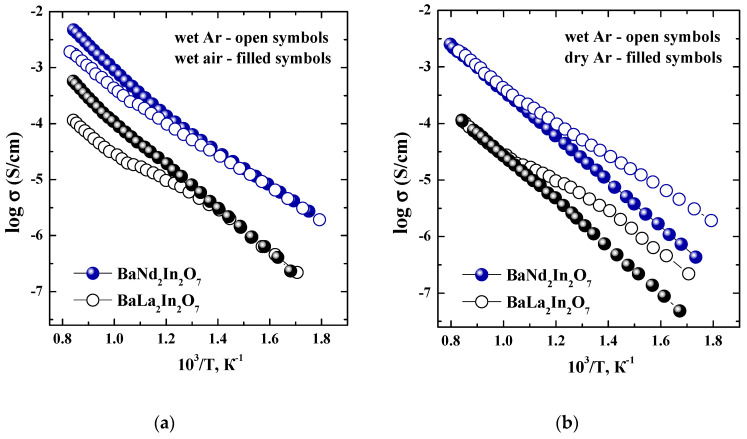
Temperature dependencies of conductivity for BaNd_2_In_2_O_7_ (blue symbols) and BaLa_2_In_2_O_7_ (black symbols) [17] obtained at wet Ar (open symbols) and wet air (filled symbols) (**a**); wet Ar (open symbols) and dry Ar (filled symbols) (**b**).

**Figure 8:**
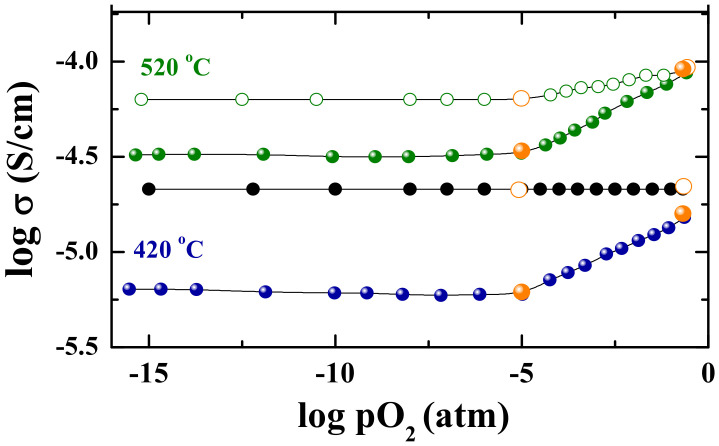
Oxygen partial pressure dependencies of the total conductivity for the BaNd_2_In_2_O_7_ sample at dry (filled symbols) and wet (open symbols) conditions and conductivity values from *σ*–10^3^/*T* dependencies (orange symbols).

The temperature dependencies of conductivity obtained under wet condition are presented in Figure 7a (*p*H_2_O = 2 × 10^−2^ atm). The conductivity values for the composition BaNd_2_In_2_O_7_ are higher compared with the values for the composition BaLa_2_In_2_O_7_. In the region of high temperatures (T > 700 °C), where protonic transport is negligible, the conductivity values obtained under wet Ar are significantly lower than under wet air. In the region of low temperatures (T < 450 °C), the conductivity values obtained under wet air and wet Ar are almost the same. In other words, the conductivity values obtained under wet air below 450 °C can be considered as the values of ionic conductivity. This statement is proved by the data obtained from the “σ–*p*O_2_” dependencies. As can be seen in Figure 8, the conductivity values obtained under wet condition and low temperatures (T < 450 °C) are independent of the oxygen partial pressure even under oxidizing conditions (*p*O_2_ = 10^−5^–0.21 atm).

The effect of changes in the water partial pressure on the ionic conductivity is well visible in Figure 7b, where temperature dependencies of conductivity under dry and wet Ar are presented. As can be seen, the decrease in the temperature leads to the increase in the conductivity values obtained under wet Ar due to formation of the protonic charge carriers in the structure, which are more mobile in comparison with oxygen ions.

**Figure 9:**
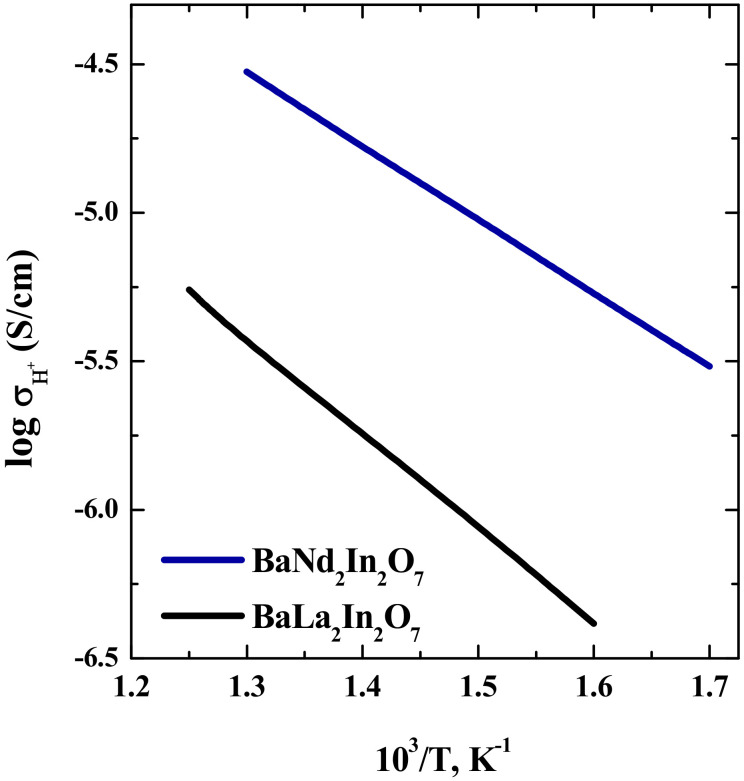
Temperature dependencies of protonic conductivity for BaNd_2_In_2_O_7_ (blue line) and BaLa_2_In_2_O_7_ (black line).

The protonic conductivity values were calculated as difference between the conductivity values in wet and dry Ar. The temperature dependencies of protonic conductivities for the compositions BaLa_2_In_2_O_7_ and BaNd_2_In_2_O_7_ are presented in Figure 9.

As can be seen, the values for BaNd_2_In_2_O_7_ are higher than for BaLa_2_In_2_O_7_ about one order of magnitude. The increase in the protonic conductivity values is well correlated with the decrease in the values of activation energy from 0.63 eV for BaLa_2_In_2_O_7_ to 0.5 eV for BaNd_2_In_2_O_7_. Returning to the results of TG-measurements, which showed closeness of protons concentrations for both compositions, we can say, that the increase in the protonic conductivity values for the composition BaNd_2_In_2_O_7_ is due to the increase in the protons mobility. The calculation of protonic transport numbers *t*_H_ according to the formula:

(6) $$t_{H}=\frac{\sigma_{H^{+}}}{\sigma_{wet\;air}}$$

This showed, that for both compositions BaLa_2_In_2_O_7_ and BaNd_2_In_2_O_7_ with decreasing temperature the increase in *t*_H_ occurs and the values reach up to 95% at the temperatures below 350 °C. Thus, these complex oxides can be considered as promising matrix compositions for creation of novel high-conductive protonic electrolytes with PR structure.

## 4. Conclusions

In this paper, the complex oxides BaLa_2_In_2_O_7_ and BaNd_2_In_2_O_7_ were synthesized, and the local structure, water uptake, and the electrical conductivity were investigated. It was shown that the values of electrical conductivity of double-layered complex oxides with PR structure BaM_2_In_2_O_7_ (M = La, Nd) are strongly depend on the nature of rare earth metal in the cationic sublattice. The change in the ionic radius of the element leads not only to the change in the unit cell parameters but also to the change in the local structure. It was shown that the composition BaNd_2_In_2_O_7_ is characterized by the higher values of electrical conductivity and the higher share of oxygen-ionic conductivity under dry air condition compared to the composition BaLa_2_In_2_O_7_. The most likely reason for this is an increase in local distortions in the crystal lattice and the formation of a greater number of oxygen defects. Under wet air conditions both compositions are nearly pure protonic conductors below 350 °C.

## Figures and Tables

**Table 1:** Unit cell parameters and volume of the BaNd_2_In_2_O_7_ and BaLa_2_In_2_O_7_ compounds.

Composition	Unit Cell Parameters	This Work	Titov et al. [27]	Raveau et al. [28]
BaNd_2_In_2_O_7_	a, Å	5.8916(9)	5.8969(8)	5.8940(3)
	c, Å	20.469(0)	20.439(3)	20.467(1)
	V, Å^3^	710.52	710.76	711.02
BaLa_2_In_2_O_7_	a, Å	5.914(9)	5.915(2)	5.9141(3)
	c, Å	20.846(5)	20.861(1)	20.831(2)
	V, Å^3^	729.34	729.92	729.73

**Table 2:** The average metal ratios determined by EDS analysis for the BaNd_2_In_2_O_7_ and BaLa_2_In_2_O_7_ compounds (theoretical values are given in the brackets).

Element	Content of the Elements, Atomic %	
	BaNd_2_In_2_O_7_	BaLa_2_In_2_O_7_
Ba	8.81 (8.33)	8.42 (8.33)
La/Nd	16.23 (16.67)	16.37 (16.67)
In	16.95 (16.67)	16.71 (16.67)
O	58.01 (58.33)	58.50 (58.33)

**Table 3:** Wavenumbers (cm^−1^) of Raman bands for the BaNd_2_In_2_O_7_ and BaLa_2_In_2_O_7_ compounds.

No of Band	BaLa_2_In_2_O_7_	BaNd_2_In_2_O_7_
1	120	118
2	140	133
3	161	157
4	203	181
5	237	-
6	260	250
7	279	-
8	316	319
9	336	-
10	358	370
11	430	453
12	445	-
13	545	660
14	800	-

## Data Availability

Not applicable.
